# Targeting Mitochondrial Dysfunction with L-Alpha Glycerylphosphorylcholine

**DOI:** 10.1371/journal.pone.0166682

**Published:** 2016-11-18

**Authors:** Gerda Strifler, Eszter Tuboly, Anikó Görbe, Mihály Boros, Daniella Pécz, Petra Hartmann

**Affiliations:** 1 Institute of Surgical Research, University of Szeged, Szeged, Hungary; 2 Department of Biochemistry, University of Szeged, Szeged, Hungary; Universidad Pablo de Olavide, SPAIN

## Abstract

**Background:**

We hypothesized that L-alpha-glycerylphosphorylcholine (GPC), a deacylatedphosphatidylcholine derivative, can influence the mitochondrial respiratory activity and in this way, may exert tissue protective effects.

**Methods:**

Rat liver mitochondria were examined with high-resolution respirometry to analyze the effects of GPC on the electron transport chain in normoxic and anoxic conditions. Besides, Sprague-Dawley rats were subjected to sham operation or standardized liver ischemia-reperfusion (IR), with or without GPC administration. The reduced glutathione (GSH) and oxidized glutathione disulfide (GSSG), the tissue myeloperoxidase, xanthine oxidoreductase and NADPH oxidases activities were measured. Tissue malondialdehyde and nitrite/nitrate formation, together with blood superoxide and hydrogen-peroxide production were assessed.

**Results:**

GPC increased the efficacy of complex I-linked mitochondrial oxygen consumption, with significantly lower in vitro leak respiration. Mechanistically, liver IR injury was accompanied by deteriorated mitochondrial respiration and enhanced ROS production and, as a consequence, by significantly increased inflammatory enzyme activities. GPC administration decreased the inflammatory activation in line with the reduced oxidative and nitrosative stress markers.

**Conclusion:**

GPC, by preserving the mitochondrial complex I function respiration, reduced the biochemical signs of oxidative stress after an IR episode. This suggests that GPC is a mitochondria-targeted compound that indirectly suppresses the activity of major intracellular superoxide-generating enzymes.

## Introduction

Ischemia-reperfusion (IR) injury is a common complication of inflow-controlled major surgical resections and organ transplantations. The prolonged lack of oxygen during ischemia is accompanied by an inevitable decrease in ATP production and an increase in ATP hydrolysis, while the overproduction of reactive oxygen and nitrogen species (ROS and RNS, respectively) during the reoxygenation phase leads to oxidative and nitrosative stress and membrane function failure. In addition to these events, the IR-induced increased activity of the main lipolytic enzymes also results in modified biomembrane structures, leading to a loss of essential membrane-forming glycerophospholipids[1, 2].

These reactions can jointly influence the function of the inner mitochondrial membrane, which embeds the four major respiratory chain complexes and the F_O_F_1_-ATP synthase (complex V) of the electron transport chain (ETC) [3, 4]. Interestingly, mitochondrial inhibitors of the oxidative phosphorylation (OxPhos) system can directly increase phosphatidylcholine (PC) breakdown by activating phospholipase A_2_, leading to an increased concentration of metabolic products [5]. It has also been demonstrated that membrane PC is depleted after an IR insult, and the liberated choline can play a protective role in the intracellular redox imbalance [6]. Furthermore, it was also shown that hepatic concentrations of glycerylphosphocholine (GPC) are significantly reduced after a period of hemorrhagic shock, with recovery to the baseline only 48 h later [7]. L-alpha-GPC is a water-soluble deacylated metabolite of PC [8], a source of choline and precursor of acetylcholine [9, 10]. Under physiological conditions, GPC can be involved in the preservation of the structural integrity of the cellular membranes, probably through the stimulation of PC synthesis via the Kennedy pathway [11]. Earlier studies from our laboratory have demonstrated that GPC administration can reduce several signs of oxidative and inflammatory tissue damage in experimental IR models [12, 13]. The anti-inflammatory action of a potentially parasympathomimetic compound is an interesting finding, because that targets the inflammatory cascade without the confounding effects of mediators deriving from the metabolism of the lipid side-chains [14].

From therapeutic aspects, influencing mitochondrial damage is appropriate strategy in hypoxia- or IR-related conditions, and the above indirect evidences all suggest that GPC may be an active and efficient compound in this setting. Based on this hypothesis we designed *in vitro* tests using intact liver mitochondria and high resolution respirometry to analyse the effects of GPC on mitochondrial function and on hypoxia-induced dysfunction. Then, we investigated the *in vivo* functional changes of the liver mitochondria in response to a standardized IR challenge. We also hypothesized that if the protective mechanism of GPC is linked to mitochondria, this mechanism of action will interfere with the ETC dysfunction-caused ROS generation, and influence the pro-inflammatory cellular activation as well.

## Materials and Methods

The experiments were carried out on male Sprague-Dawley rats (average weight: 300±20 g, 7–8 weeks old) housed in an environmentally controlled room with a 12-h light-dark cycle, and kept on commercial rat chow (Standard rat chow LT/n; InnovoKft, Gödöllő, Hungary) and tap water ad libitum. The experimental protocol was in accordance with EU directive 2010/63 for the protection of animals used for scientific purposes, and it was approved by the National Scientific Ethical Committee on Animal Experimentation (National Competent Authority) with the license number V./148/2013. This study also complied with the criteria of the US National Institutes of Health Guidelines for the Care and Use of Laboratory Animals.

### In vitro experimental protocol

We have performed *in vitro* experiments to detect the changes in the respiratoryactivity of liver mitochondria in response to 30-min anoxia, with or without GPC administration, using high-resolution respirometry (Oxygraph-2k, Oroboros Instruments, Innsbruck, Austria). In this experimental series, the animals were anesthetized for sample taking using sodium pentobarbital (45 mg/kg ip). The liver biopsy samples were homogenized in 1 ml of MitOx respiration medium [15] with a glass Potter homogenizer. Subsequently, the homogenates were weighed into the detection chambers, 50 μl in each, which were calibrated to 200 nmol/ml oxygen concentration in room air. In order to determine the effective GPC concentration range, series of GPC solutions from 1 nM to 800 mM were used. The steady-state basal oxygen consumption of the homogenates (respiratory flux) were measured. The complex II-linked state II respiration rate was then determined with 10 mM succinate after the addition of 0.5 μM complex I inhibitor rotenone. Then the complex II-linked (state III respiration) maximum respiratory capacity was estimated by adding saturating concentration of ADP to the medium. Subsequently, anoxia was applied and, at the end of the 30-min anoxic period, the chambers were opened to recover the mitochondria at 200 nmol/ml oxygen concentration.

### In vivo experimental protocol

In the *in vivo* experimental series, the animals were anesthetized with sodium pentobarbital (45 mg/kg ip), and the trachea was cannulated to facilitate respiration. The right jugular vein and carotid artery were cannulated for fluid and drug administration, respectively. Further small supplementary doses of pentobarbital were given intravenously when necessary. The animals were placed in a supine position on a heating pad to maintain the body temperature between 36 and 37°C, and Ringer's lactate was infused at a rate of 10 ml/kg/h during the experiments. After midline laparotomy and bilateral subcostal incisions, the liver was carefully mobilized from all ligamentous attachments, and complete ischemia of the median and left hepatic lobes was achieved by clamping the left lateral branches of the hepatic artery and of the portal vein with a microsurgical clip for 60 min [16, 17]. In this model ischemia involves approx. 70% of the liver, while the circulation of the right liver lobe remains intact, providing blood flow towards the heart and thus avoiding hepatic congestion. After ischemia, the clips were removed and the wound was temporarily covered with non-water-permeable foil during the 60-min reperfusion period. At the end of the experiments the animals were over-anesthetized with a single overdose of pentobarbital.

The animals were randomly assigned to four groups. In the vehicle-treated IR group (n = 6), the rats were subjected to a 60-min complete ischemia followed by a 60-min reperfusion; in the IR+GPC group a 16.56 mg/kg bw GPC (MW: 257.2, Lipoid GmbH, Ludwigshafen, Germany; dissolved in 0.5 ml of sterile saline solution at 0.064 mM concentration) was injected intravenously and the same protocol was used [12], 5 min before the end of ischemia. The sham-operated, vehicle-treated animals (SHAM group, n = 6) underwent the same surgical procedure without liver ischemia, while another control group (SHAM+GPC group, n = 6) received GPC in the same time-frame as the IR+GPC group.

#### The Substrate-Uncoupler-Inhibitor Titration (SUIT) protocol

To measure the respiratory activity of the liver mitochondria, tissue samples were homogenized in mitochondrial respiration medium and then subjected to high-resolution respirometry. The SUIT protocol was employed to explore the relative contribution of complex I (C-I) and complex II (C-II) to the electron transport system. Glutamate (2 mM) and malate (10 mM) were used in combination to induce C-I-linked respiration, saturating ADP (2.5 mM final concentration) was added in order to stimulate respiration to the level of OxPhos capacity. By adding succinate (10 mM), the C-I+C-II OxPhos capacity was detected, then the uncoupler carbonyl cyanide m-chlorophenyl hydrazine (CCCP) (C; 0.5 μM per step) was titrated. Finally, C-I was inhibited by rotenone (0.5 μM) and C-III by antimycinA (2.5 μM).

#### The Leak protocol

In order to determine the leak respiration, liver samples were homogenized in 1 ml of MitOx medium, then 50 μl of homogenates were weighed into the detection chambers. The complex II-linked state II respiration rate was then determined with 10 mM succinate, after the addition of 0.5 μM complex I inhibitor rotenone. To determine the complex II-linked stateIII respiration, 2.5 mM ADP was added to each chamber. Finally, the leak respiration is measured in the leak state by inhibition of ATP synthase by adding 0.5 μMoligomycin to the medium (state IV respiration).

#### Tissue xanthine oxidoreductase (XOR) activity

Liver biopsies were homogenized in phosphate buffer (pH 7.4) containing 50 mMTris-HCl, 0.1 mM EDTA, 0.5 mMdithiotreitol, 1 mMphenylmethylsulfonyl fluoride, 10 μg ml^−1^ soybean trypsin inhibitor, and 10 μg ml^−1^leupeptin. The homogenate was centrifuged at 4°C for 20 min at 24.000 g, and the supernatant was loaded into centrifugal concentrator tubes. The activity of XOR was determined in the ultrafiltered supernatant by a fluorometric kinetic assay based on the conversion of pterine to isoxanthopterine in the presence (total XOR) or absence (XO activity) of the electron acceptor methylene blue [18].

#### NADPH oxidase activity

The NADPH oxidase activity of the liver homogenates was determined by a modified chemiluminometric method of Bencsik et al. [19]. The Liver samples were homogenized in 2 ml MitOx medium, then 50 μl of resuspended homogenate was added in Dulbecco’s solution containing lucigenin (10 mM), EGTA (10 mM) and saccharose (900 mM). The NADPH oxidase activity was determined via the NADPH-dependent increase in luminescence elicited by adding 1 mM NADPH (in 20 μl), measured with an FB12 Single Tube Luminometer(Berthold Detection Systems GmbH, Bad Wildbad, Germany). Samples incubated in the presence of nitrobluetetrazolium served as controls. The measurements were performed intriplicates and were normalized for protein content. The protein content of the samples was determined with Lowry’s method.

#### Reduced glutathione and oxidized glutathione disulfide (GSH/GSSG) ratio in liver homogenates

The reduced glutathione (GSH) and oxidized glutathione disulfide (GSSG) ratio was determined by using a FluorimetricGluthatione Assay Kit (Sigma Aldrich, Budapest, Hungary). The GSH content of the sample can be determined by quantifying the thiol concentration in biological samples by reacting with the thiol groups they contain. The adduct can be detected with fluorimetry at 478 nm. The GSSG content of the sample was calculated following the recommendations of the manufacturer.

#### Tissue myeloperoxidase (MPO) activity

The MPO activity was measured in liver biopsies by the method of Kuebler et al [20]. Briefly, the tissue was homogenized with Tris-HCl buffer (0.1 M, pH 7.4) containing 0.1 M polymethylsulfonyl fluoride to block tissue proteases, and then centrifuged at 4°C for 20 min at 24.000 g. The MPO activities of the samples were measured at 450 nm (UV-1601 spectrophotometer; Shimadzu, Japan), and the data were referred to the protein content.

#### Liver nitrite/nitrate (NOx) levels

The levels of NOx, the stable end products of NO, in the tissues were measured using the Griess reaction. This assay is based on the enzymatic reduction of nitrate to nitrite, which is then converted into a coloured azo compound, which is detected spectrophotometrically at 540 nm [21].

#### Tissue malondialdehyde (MDA) assay

The degree of lipid peroxidation was estimated via the amount of MDA, a marker of oxidative damage of lipid membranes. The MDA level was measured by the reaction with thiobarbituric acid, and the values were corrected for the tissue protein content [22].

#### Superoxide and hydrogen peroxide production in whole blood

A 10 μl sample of whole blood and 50 μlzymosan were added to 1 ml Hank’s solution (PAA Cell Culture, Westborough, MA, USA) and the mixture was incubated at 37°C for 30 min, until assay [23]. The chemiluminometric response was measured with a Lumat LB9507 luminometer (Berthold Technologies, Wildbad, Germany) during a 30-min period after the addition of 100 μl of lucigenin and luminol reagent.

#### In vivo histology

In a separate series fluorescence confocal laser scanning endomicroscopy (CLSEM, Five1, Optiscan Pty. Ltd., Melbourne, Victoria, Australia, excitation wavelength 488 nm; emission detected at 505–585 nm) developed for in vivo histology was employed to detect the extent of tissue injury in the left liver lobe. The treatments (n = 6, each group) were identical to the previous in vivo protocol.

The microvascular structure was recorded after the iv administration of fluorescein isothiocyanate-dextran (FITC-dextran, 150 KDa, Sigma-Aldrich, Budapest, Hungary, 10 mg/ml solution dissolved in saline). For the in vivo staining of liver cells, 0.01% acriflavine (Sigma-Aldrich, Budapest, Hungary) was injected into the jugular vein [24]. The objective of the device was placed onto the liver surface, and confocal imaging was performed 5 min after dye administration (1 scan/image, 1024 x 512 pixels and 475 x 475 μm per image).

The analysis was performed twice separately by the same investigator (PH) using a semiquantitative histology score (S0-S4) based on hepatocyte swelling, shrinkage, loss of integrity of cellular and nuclear membranes, or nuclear alterations, as described previously [24, 25].

#### Statistical analysis

Data analysis was performed with SigmaStat statistical software (Jandel Corporation, San Rafael, CA, USA). Changes in variables within and between groups were analysed by two-way repeated measures ANOVA, followed by the Bonferroni test. One-way ANOVA followed by the Holm-Sidak test was applied in the assay of tissue MDA, XOR activity, MPO activity, NADPH-oxidase activity, tissue nitrite/nitrate, reduced and oxidized glutathione (GSH/GSSG) ratio on liver tissue, hydrogen peroxide level and superoxide level. Data were expressed as means ± SEM. Values of P < 0.05 were considered statistically significant.

## Results

### In vitro experiments

**Fig 1:**
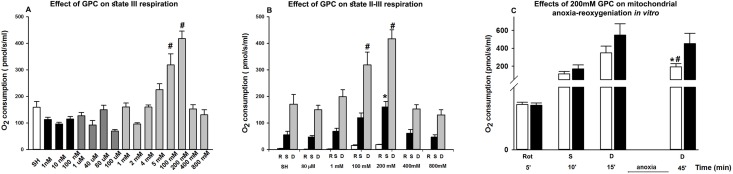
Oxygen consumption (in pmol/s/ml) of liver mitochondria measured by means of high-resolution respirometry. Liver homogenates were harvested from sham-operated animals. (A) Effect of different GPC concentrations on state III respiration of liver mitochondria. Data are means ± SEM. ^#^P< 0.05 vs SHAM group (one-way ANOVA, Holm-Sidak test). (B) Effect of GPC on state II and III respiration. ^#^P< 0.05 vs SHAM (state II) group; ^*^P< 0.05 vs SHAM (state III) group (one-way ANOVA, Holm-Sidak test). (C) Effect of 200 mM GPC on mitochondrial anoxia-reoxygenation *in vitro*. Liver homogenates were subjected to 30’ anoxia in the presence of 200 mM GPC (black column: SHAM+GPC group) or without GPC pre-treatment (white column: SHAM group). Data are presented as means ± SEM. ^#^P< 0.05 vs 5’; ^*^P< 0.05 vs SHAM group (two-way ANOVA, Bonferroni test). R: Rotenone; S: Succinate; D: ADP

Firstly, *in vitro* experiments were conducted in order to analyse the dose-response effects of GPC on the respiratory activity of rat liver mitochondria in normoxia or anoxicconditions. GPC had an increasing effect on mitochondrial oxygen consumption in the 100–200 mM concentration ranges (Fig 1A). The ETC and OxPhos capacity of mitochondria was influenced significantly when GPC was applied at 200 mM concentration (Fig 1B). In addition, GPC significantly attenuated the deleterious effects of 30-min anoxia on the oxygen consumption of mitochondria (Fig 1C).

### In vivo experiments

#### Mitochondrial respiration detected by the SUIT and Leak protocols

**Fig 2:**
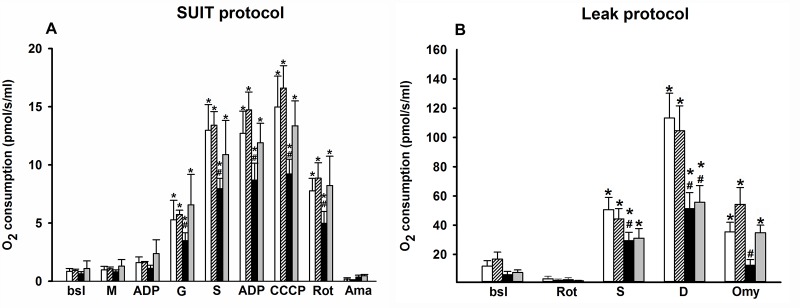
Oxygen consumption of liver mitochondria measured by means of high-resolution respirometry (in pmol/s/ml). (A) SUIT protocol. (B) Leak protocol. Animals were subjected to 60 min of liver ischemia followed by 60 min of reperfusion (IR group, black column) or were sham-operated (SHAM group, white column). 16.56 mg/kg GPC administration was started 5 min before the end of ischemia (IR+GPC group, grey column), or at identical time point in sham-operated animals (SHAM+GPC group, white striated column). Data are presented as means ± SEM. ^#^P< 0.05 vs SH group; ^*^P< 0.05 vs baseline (two-way ANOVA, Bonferroni test). **bsl: baseline; M: Malate; D: ADP; G: Glutamate; S:Succinate; CCCP: chemical inhibitor of OxPhos (uncoupler); Rot: Rotenone; Ama: Antimycin A; Omy: Olygomycin.**

The SUIT protocol provides an opportunity to analyse the activity of different mitochondrial complexes, while the Leak protocol observes the proton leak in mitochondria. In the SUIT protocol (Fig 2A), the state III oxygen consumption was significantly lower in IR compared to the sham-operated animals. Additionally, the maximum respiratory capacity was also significantly lower in response to the IR stress. In contrast, treatment with GPC enhanced the efficacy of oxygen consumption. These effects were basically linked to the complex I, rather than complex II, as indicated by the large decrease following the administration of the inhibitor of complex I.

The Leak protocol (Fig 2B) demonstrated significant decrease in state IV oxygen consumption in response to IR injury as compared to the sham-operated animals. GPC administration restored the level of leak respiration to that of the sham-operated animals.

#### XOR activity

**Fig 3:**
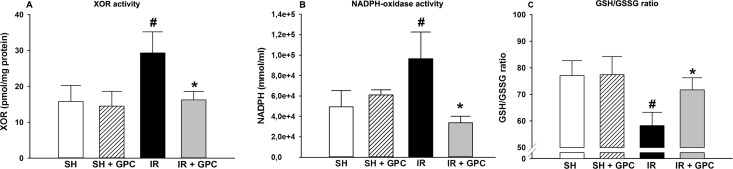
(A) XOR activity; (B) NADPH oxidasesactivity; (C) GSH/GSSG ratio. Animalsweresubjectedto 60 min of liverischemiafollowedby 60 min of reperfusion (IR group, blackcolumn) orweresham-operated (SHAM group, whitecolumn). 16.56 mg/kg GPC administrationwasstarted 5 min beforethe end of ischemia (IR+GPC group, greycolumn), oratidenticaltimepointinsham-operatedanimals (SHAM+GPC group, whitestriatedcolumn). XOR activity (inpmol/mg protein); NADPH oxidasesactivity (inmmol/ml). Data arepresentedasmeans ± SEM. #P< 0.05 vs SHAM group; *P< 0.05 vs IR group (one-way ANOVA, Holm-Sidak test).

XOR is a key enzyme in purine catabolism, and also catalyses the reduction of nitrates and nitrites into nitric oxide (NO). During this process, ROS are produced, which can be deleterious to the cells. As expected, XOR activity was increased in the IR group compared to the SHAM group. These values were significantly decreased when GPC was applied 5 min before the reperfusion (Fig 3A).

#### NADPH oxidase activity

NADPH oxidases are a family of membrane-bound oxidoreductase complexes whose main function is the formation of ROS, by catalysing the reduction of oxygen (2NADPH +2O_2_ —> 2NADP^+^ + 2H^+^ + 2O_2_- —> 2NADP^+^ + H_2_O_2_). While their precise role in the IR pathogenesis is not fully elucidated, it is assumed that NADPH oxidases play a key role in the propagation of oxidative stress. By the end of the 60-min reperfusion period, the NADPH oxidase activity was significantly increased in the IR group, compared to the SHAM groups (Fig 3B). When GPC was administered before the end of ischemia the NADPH oxidase activity became even lower than the values of the SHAM groups.

#### GSH/(GSSG) ratio in liver homogenates

GPC administration in the SHAM+GPC group did not influence the GSH/GSSG ratio as compared with the SHAM group. As expected, hepatocytes were exposed to increased levels of oxidative stress after IR, as shown by a significant increase of GSSG and the decreased GSH/GSSG ratio when compared to the SHAM group, however, the GSSG levels were significantly decreased in response to GPC treatment in the IR+GPC group (Fig 3C).

#### MPO activity

**Fig 4:**
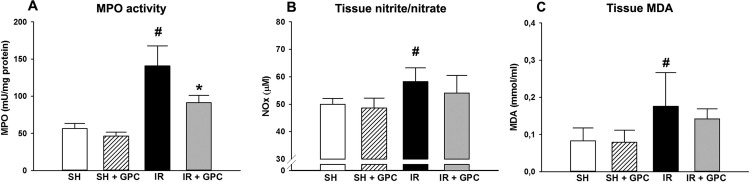
(A) Tissue MPO activity; (B) Tissue nitrite/nitrate (NOx) level; (C) Tissue MDA level. Animals were subjected to 60 min of liver ischemia followed by 60 min of reperfusion (IR group, black column) or were sham-operated (SHAM group, white column). 16.56 mg/kg GPC administration was started 5 min before the end of ischemia (IR+GPC group, grey column), or at identical time point in sham-operated animals (SHAM+GPC group, white striated column).MPO activity (in mU/mg protein); NOx level (in μM); tissue MDA (in mmol/ml). Data are presented as means ± SEM. ^#^P< 0.05 vs SHAM group; (one-way ANOVA, Holm-Sidak test).

MPO is mostly produced by PMN leukocytes upon their activation. In the vehicle-treated IR group, the tissue MPO level was significantly increased as compared with that of the sham-operated animals. In the GPC-treated group, the MPO activity was significantly lower than in the vehicle-treated IR group (Fig 4A).

#### Liver NOx levels

In the IR group, a significant elevation in NOx was present relative to the SHAM groups. The GPC treatment protocol decreased the NOx elevation, in contrast with the non-treated IR group; but the NOx level remained significantly higher than that in the sham-operated group (Fig 4B).

#### Tissue MDA level

As expected, IR resulted in an increased MDA production after IR (Fig 4C). The GPC treatment significantly reduced the level of MDA production, while no difference was seen between the two control groups (SHAM and SHAM+GPC).

#### Blood hydrogen peroxide and superoxide production

**Fig 5:**
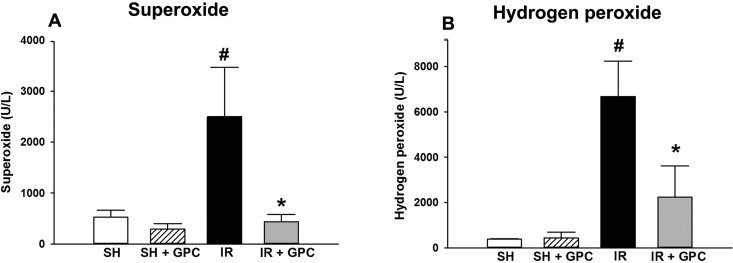
(A) Superoxide level; (B) Hydrogen peroxide level. Animals were subjected to 60 min of liver ischemia followed by 60 min of reperfusion (IR group, black column) or were sham-operated (SHAM group, white column). 16.56 mg/kg GPC administration was started 5 min before the end of ischemia (IR+GPC group, grey column), or at identical time point in sham-operated animals (SHAM+GPC group, white striated column). Superoxide level (in U/L); hydrogen peroxide level (in U/L). Data are presented as means ± SEM. ^#^P< 0.05 vs SHAM group; ^*^P< 0.05 vs IR group (one-way ANOVA, Holm-Sidak test).

The superoxide-producing capacity in the whole blood was significantly higher in the IR group at the end of reperfusion when compared to the SHAM animals. The GPC treatment before the end of the ischemic period reduced the elevated superoxide production to the level in the control animals (Fig 5B). Significantly higher whole blood hydrogen peroxide levels were measured at the end of reperfusion in the IR group relative to the SHAM group, and the GPC treatment effectively reversed the hydrogen peroxide production (Fig 5A).

#### In vivo histology

**Fig 6:**
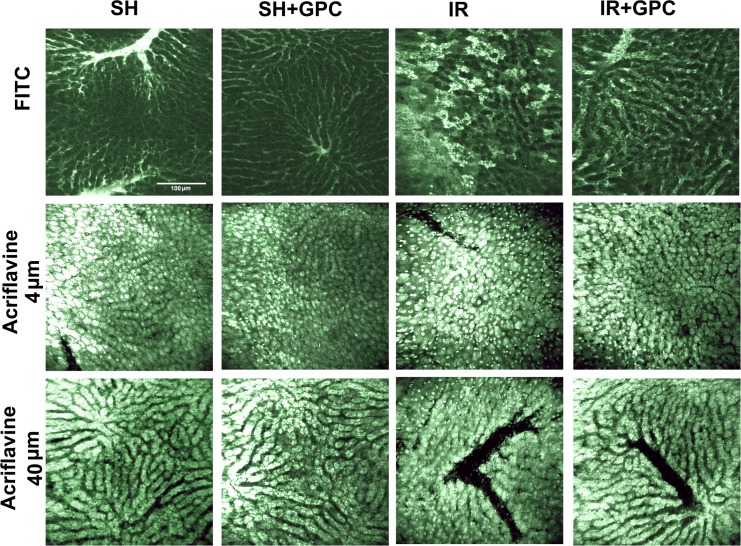
Histological changes in the rat liver. Tissue sections show the results of in vivo fluorescence confocal laser scanning endomicroscopy (CLSEM) with FITC dextran and acriflavine labelling (at 4 and 40 μm depth). Structural damages such as dilated sinusoids, loss of fluorescence intensity, changes in hexagonal cell shape, cytoplasmaticblebbing and vesicle formation can be observed in the IR group. The bar represents 100 μm.

The morphological changes in the left liver lobe were evaluated by means of in vivo imaging, using CLSEM. The FITC-dextran and acriflavine staining demonstrated dilated sinusoids in the IR group, fluorescent dye leakage with edema formation was present with visible signs of structural damage: changes in hexagonal cell shape and cytoplasm blebbing and vesicle formation. GPC administration effectively attenuated the IR-induced morphological changes. The severity of injury was moderated, these changes were still apparent, but the average degree of damage was decreased from S4 to S2 level (Fig 6).

## Discussion

IR injury is a common challenge of several fields of medicine. IR-induced antigen-independent inflammatory reactions are largely ignited by the overproduction of ROS and, mainly at the sites of complexes I and III, the mitochondria is among the major recognized sources [26]. Therefore, targeted therapeutic strategies to limit mitochondrial dysfunction and to tackle the potentially cell-damaging consequences of ROS generation are important translational research tasks.

GPC is a choline donor compound with a demonstrated parasympathomimetic action [11]. It is currently used in clinical practice to enhance functional recovery after cerebral stroke [10], and to reduce the cognitive symptoms of dementia [27, 12]. Exogenous GPC is detected in the circulation one hour after an iv injection, it passes through the blood-brain barrier, stimulates PC biosynthesis, and activates postsynaptic cholinergic receptors [9, 13, 28]. Tissue distribution studies have shown that GPC and its metabolites are particularly accumulated in organs of excretion (kidney/liver), but the liver contains the highest concentration [9, 13]. Cellular uptake of GPC may occur by osmoregulation or under the control of an active transport system [29]. Osmotic regulation of GPC requires choline in the medium, presumably as a precursor for GPC synthesis. Choline transport into the cells, however, is not osmoregulated. Furthermore, an increase in GPC content under hyperosmotic conditions is not associated with an increased activity of the transport systems of biosynthetic precursors [30]. These results suggest some interaction between the two regulatory systems in the cellular uptake of the circulating GPC.

Regardless of its possible abundance in the membranes, liver concentrations of endogenous GPC are significantly depleted after hemorrhagic shock, a prototype of systemic IR injury [7], and previous data suggested us that exogenous GPC may influence the tissue reactions in IR scenarios [12, 13, 31]. Herein, we have outlined a novel route of action for the molecule. The present study explains the anti-inflammatory action at mitochondrial level and provides a mechanistic basis for these observations.

The in vitro experimental data demonstrated the direct effects of GPC on mitochondrial oxygen consumption in the 100 and 200 mM concentration ranges. Next, GPC supplementation attenuated the respiratory consequences of anoxia by reducing the leak of protons into the matrix and preserving the state III respiration of mitochondria. This effect was mainly attributed to an action on complex I at the appropriate concentration of 200 mM. Within the mitochondria, the mechanism by which GPC increases basal oxygen consumption rates is not well-understood, but the possibilities can be listed in two categories: 1) interacting with proteins and causing modulations in their functions or 2) influencing the redox environment. It should be added that the I-V sequence of the respiratory complexes is perhaps not the highest level of OxPhos organization. Flux control experiments confirm that the respiratory chain operates as one single functional unit [32, 33]. According to the “fluid-state model”, individual protein complexes of the electron transport chain freely diffuse in the membrane and the electron transfer is based on random collisions of single complexes. Recent findings also suggest that OxPhos enzymes are organized into supramolecular assemblies[34]. It has been shown that point mutations in genes of the subunits of an OxPhos complex affect the stability of another complex. Thus, complex III and complex IV are necessary for the assembly or stability of complex I [34, 35]. Moreover, it appears that supercomplexes are further organized into larger string structures. The example is the ATP synthase complex (complex V), which assembles into long oligomeric chains [36]. Some supercomplexes require appropriate osmotic environment for their formation [37, 38]. Whether GPC influences the conformation of this system is an open question.

Secondly, the redox-optimized ROS balance hypothesis postulates that the redox environment is the main controller of both production and scavenging of ROS as intermediary between mitochondrial respiration and ROS formation [39]. We have shown that exogenous GPC targets the mitochondrial oxidative metabolism in IR stress, and provided evidence that the IR-associated inflammatory activation may be limited this way. Mitochondrial dysfunction generates ROS and hypoxic conditions induce leak of protons of the ETC into the intermembranous space [40] that can lead to increased ROS formation. We have demonstrated that GPC treatment reduces the leak respiration after the IR challenge, and in accordance with previous findings the lower leak respiration was accompanied with decreased ROS formation [7, 40, 41]. Furthermore, exogenous GPC enhanced mitochondrial oxygen consumption, both in normoxic and hypoxic conditions, which clearly demonstrates that GPC can potentiate the mitochondrial activity. To further clarify this issue, another protocol was applied by adding substrates and inhibitors of individual ETC complexes. In response to complex I inhibitor rotenone, the oxygen consumption diminished significantly, which suggests that complex I is the target site of the GPC-mediated action.

We have investigated IR-induced ETC changes together with XOR and NADPH oxidases responses. The activity of both pro-inflammatory enzymes were decreased in response to GPC administration, which suggests that the primary influence on leak respiration was followed by secondary consequences on the main extra-mitochondrial, i.e. cellular enzymes involved in ROS formation. Furthermore, the IR-induced increases in superoxide and hydrogen peroxide levels in the circulating blood were accompanied by increased local NOx concentrations, providing indirect evidence for the evolving oxido-nitrosative stress in the liver tissue. RNS acting together with ROS generates “footprints” of tissue damage [42]. ROS interacting with NO produces peroxynitrite [43] and nitration of mitochondrial proteins resulting in acute and chronic liver diseases.

In our model, the increase in MDA and other oxidative and nitrosative stress markers were significantly reduced by GPC supplementation. The need of restoration of cellular GSH levels for efficient scavenging of peroxynitrite is emphasized. GPC administration reversed the IR-induced decrease in GSH level and maintained the ratio of GSH to GSSG.

We also detected increased MPO activity as a secondary inflammatory marker, mainly secreted by active immune cells including PMNs. Again, MPO activity was decreased after the administration of GPC. All considered, these results suggest that mitochondrial alterations preceded the cellular, enzymatic ROS production and the onset of oxidative stress in liver tissue lead to PMN activation in the circulation.

In conclusion, we have shown that exogenous GPC influences the mitochondrial oxidative metabolism, the primary source of ROS production. Nevertheless, this study has some limitations which have to be pointed out. Firstly, the isolation of mitochondria can disrupt the intricate mitochondrial network integrity leading to possible differences between in vitro and in vivo data. In this line, the impact of cytosolic factors could not be correctly studied due to the loss of cytoplasm and the cellular soluble components during the isolation procedure. This is of particular importance because the normal mitochondrial function requires a crosstalk with cytosolic factors (such as the GSH/GSSG system) to maintain cellular homeostasis, and the in vivo experiments have been conducted in the absence of scavengers that control glutathione levels. Besides, the assessment of mitochondrial respiration was performed without measuring the protein levels of individual respiratory enzyme complexes, thus a possibility that altered complex levels contributed to the in vivo respirometry changes cannot be excluded.

However, we have provided evidence for the direct action of GPC on mitochondrial complex I function as proved by increased oxygen consumption and the reduced leak respiration. The redox-imbalance of the intracellular environment, altered enzyme activities can lead to further increases in superoxide production. GPC administration attenuated the membrane peroxidation and the consecutive stages of tissue damage therefore this type of mechanism might be an interesting focus for therapeutic strategies in IR episodes.

## Supporting Information

S1 Fig Hydrogen peroxide (H2O2) production (pmol/s/mg protein) of liver mitochondria measured by means of high-resolution respirometry. Animals were subjected to 60 min of liver ischemia followed by 60 min of reperfusion (IR group, black column) or were sham-operated (SHAM group, white column). 16.56 mg/kg GPC administration was started 5 min before the end of ischemia (IR+GPC group, grey column), or at identical time point in sham-operated animals (SHAM+GPC group, white striated column). Data are presented as means ± SEM. #P< 0.05 vs SH group; *P< 0.05 vs baseline (two-way ANOVA, Bonferroni test). Rot: Rotenone; Ama: Antimycin A; FCCP: Carbonyl cyanide p-trifluoro-methoxyphenylhydrazone, uncoupler.
(TIF)

S2 Fig Complex I and Complex II-dependent oxygen consumption (in pmol/s/ml) of isolated liver mitochondria measured by means of high-resolution respirometry. Mitochondria were subjected to 30-min anoxia and 30-min reoxygenation (AR group, black column) or AR and 200 μmol GPC pretreatment (AR+GPC, grey column). Mitochondria in normoxic environment served as controls (SH and SH+GPC, white and striated columns). Data are presented as means ± SEM. *P< 0.05 vs AR group (one-way ANOVA, Holm-Sidak test).
(TIF)

S1 File Experimental protocols and results of additional experimental series: Examination of reverse electron transport (RET) and Examination of Complex I and II–linked respiration. (PDF)
