# Supplementary material for: Targeting Mitochondrial Dysfunction with L-Alpha Glycerylphosphorylcholine
Source: PLoS One. 2016 Nov 18;11(11):e0166682. doi: 10.1371/journal.pone.0166682 (PMC5115775; doi:10.1371/journal.pone.0166682)
Supplement: S1 File — (PDF) [file pone.0166682.s003.pdf]

S1 File. Experimental protocols and results of additional experimental series: Examination of reverse electron transport (RET) and Examination of Complex I and II–linked respiration.

EXAMINATION OF REVERSE ELECTRON TRANSPORT (RET)

We have performed additional *in vivo* experiments for the direct examination of RET independently to the forward electron flux in rat liver mitochondria. In this series, the mitochondrial hydrogen peroxide (H_2O_2) release as marker of ROS (i.e. superoxide anion) production was monitored fluorimetrically by the Amplex Red/horseradish peroxidase system, whereby Amplex Red (non-fluorescent) is oxidized to Resorufin. Calibration of H_2O_2 production was performed by known amounts of H_2O_2 and ROS release was investigated with oxidizing complex II-substrate succinate in the presence of respiratory chain inhibitors and protonophore FCCP.

RESULTS

In the SH group, ROS generation with succinate as the sole substrate was strongly inhibited by rotenone and was partly, but not fully, restored by a subsequent addition of antimycin A (S1 Fig.). The ROS generation associated with succinate oxidation in the absence of respiratory chain inhibitors is very sensitive to depolarization of the inner membrane. Thus, ROS release was strongly decreased by the protonophoric uncoupler FCCP. In response to IR, the RET-related ROS production was significantly increased, which supports the notion that the selective accumulation of the citric acid cycle intermediate succinate is a universal metabolic signature of ischaemia and at least partially responsible for mitochondrial ROS production during reperfusion. When mapping the sites for ROS production at the level of the mitochondrial respiratory chain (i.e. complex I and complex III), it appears that ROS production linked to the reverse electron flux at complex I (succinate alone and rotenone-

sensitive) is predominant as compared to other sources linked to forward electron flux, since rotenone addition almost fully abolished this ROS production. The lower ROS production in the presence of GPC administration is in agreement with the earlier data (earlier observation in the SUIT protocol - see Figure 2).

EXAMINATION OF COMPLEX I AND II–LINKED RESPIRATION

Additional *in vitro* experiments were conducted to compare the 30-min anoxia and 30-min reoxygenation (AR) induced changes in complex I and complex II-linked respiration of isolated mitochondria with or without 200 µmol GPC administration (AR and AR+GPC groups respectively; n=8-8). Mitochondria in normoxic environment with or without GPC treatments served as controls (SH and SH+GPC respectively; n=8-8).

Rat liver mitochondria from the left liver lobe were isolated by the method of Gnaiger et al.[1]. Briefly, mitochondrial pellets were resuspended in isotonic sucrose medium (300 mM sucrose, 0.2 mM EDTA and 10 mM HEPES, adjusted to pH 7.4 with KOH at 4 °C) containing 100 µmol N-acetyl cysteine[2]. For respirometric analysis, isolated mitochondria were suspended in 3 ml MitOx2 medium and weighed into the detection chambers.

During the respirometric analysis, parallel protocols were run in the two chambers for comparison of complex I and complex II-linked oxygen flux. In chamber A, the complex I-dependent respiration was measured after the addition of complex II inhibitor malonic acid (10 mM) and 2 mM malate and 10 mM glutamate substrates. Then, saturating concentration of 5 mM ADP for complex I state III respiration was added to the medium. In chamber B, the complex II-linked respiration was determined after the addition of complex I inhibitor 0.5 µM rotenone and 10 mM succinate substrate. Finally, 5 mM ADP was administered for measuring the complex II-dependent oxidative phosphorylation (OxPhos) capacity.

RESULTS

During the complex I protocol, AR resulted in a significantly lower OxPhos capacity of the mitochondria (complex I-linked state III respiration) in comparison with the SH group. When GPC was administered, however, the respiratory capacity was reversed to the level of SH mitochondria (see S2 Fig.). In contrast, there was no significant difference in the respiratory flux between the groups when the complex II protocol was applied. Thus, neither AR, nor incubation of the respiration medium with GPC affected the function of complex II, as compared with SH mitochondria.

These results suggest that the hypoxic deactivation of complex I might initially act as an intrinsic protective mechanism against the overproduction of ROS and provide a way to recover the cellular bioenergetic function after reoxygenation. However, slower recovery of complex I can contribute to cell injury by both limiting the electron transport required for OxPhos and increasing the production of ROS. Our results demonstrate the sensitivity of complex I-linked OxPhos capacity to AR injury and the protective role of GPC to maintain the electron transport when oxygen concentration rises. The complex II-linked respiration, when investigated separately from the complex I function, was not affected by the AR-related changes.

REFERENCES

1. Gnaiger E, Kuznetsov AV, Schneeberger S, Seiler R, Brandacher G, Steurer W, Margreiter R. Mitochondria in the cold. In: Life in the Cold. New York: Springer, 2000. pp. 431-442.

2. Puranam KL, Wu G, Strittmatter WJ, Burke JR. Polyglutamine expansion inhibits respiration by increasing reactive oxygen species in isolated mitochondria. Biochem Biophys Res Commun. 2006;2:607-613.
